# Bioanalytical Methods in Toxicology

**DOI:** 10.1080/15376510802156655

**Published:** 2008-06-23

**Authors:** Elżbieta Skrzydlewska

**Affiliations:** Department of Analytical Chemistry, Medical University of Białystok 15-230 Białystok 8, P. O. Box 14, Poland

The goal of this ***Special Issue on Bioanalytical Methods in Toxicology*** is to recapitulate the latest achievements concerning the theory and practice of modern chromatography and related techniques. The interest of such significant and current issues has not been accidental. Chromatography techniques and related methods, including sample preparation in pharmaceutical and biomedical applications, have taken a lead in evaluation of food quality and of the condition of our natural environment. The methods presented in this special issue deal with separating, coupled techniques or hybrid systems, accompanied by miniaturization and development of new methods and analytical procedures, which specify the range of determinations on the vestigal or subultravestigal level. At the same time, they indicate areas of research in the contemporary analytical chemistry, which is more and more appreciated by other disciplines.

Problems addressed in the presented investigations have confirmed that continuously developed separating techniques such as gas and liquid chromatography require extensive study on the concentration of compounds contaminating food and the natural environment, including remnants of drugs. Independently of food quality testing, new methods employed for quantitative and qualitative control of diet supplements are developed intensively.

Among the presented bioanalytical data in this special issue, a wide group of studies focuses on application of chromatographic methods in analysis of biologically significant compounds and their metabolites occurring in human and animal tissues. The greatest interest was focused on the bilipids and products of their metabolism, generated in physiological conditions as well as in pathological processes including drug therapy. Most of the reports on separating methods indicate strict requirements for initial purification of samples by liquid–solid or liquid–liquid extraction.

In conclusion, it should be emphasized that continuous improvement of separating techniques and development of new analytical methods enable determination of a growing number of new compounds in a scale of nanotechnology that is an emerging and important topic in the field of toxicology.

